# Transcriptome analysis of the biofilm formation mechanism of *Vibrio parahaemolyticus* under the sub-inhibitory concentrations of copper and carbenicillin

**DOI:** 10.3389/fmicb.2023.1128166

**Published:** 2023-03-02

**Authors:** Jiaying Xie, Hongmin Zhang, Yinhui Li, Hao Li, Yingjie Pan, Yong Zhao, Qingchao Xie

**Affiliations:** ^1^College of Food Science and Technology, Shanghai Ocean University, Shanghai, China; ^2^Laboratory of Quality and Safety Risk Assessment for Aquatic Product on Storage and Preservation, Ministry of Agriculture and Rural Affairs, Shanghai, China; ^3^Shanghai Engineering Research Center of Aquatic Product Processing and Preservation, Shanghai, China

**Keywords:** *Vibrio* *parahaemolyticus*, minimal inhibitory concentration, biofilm formation, transcriptome, copper and carbenicillin

## Abstract

Biofilm formation of *Vibrio parahaemolyticus* enhanced its tolerance to the environment, but caused many serious problems to food safety and human health. In this paper, the effects of copper and carbenicillin (CARB) stress on the formation of the biofilms of *V. parahaemolyticus* organisms were studied, and RNA sequencing technology was used to compare the differences in transcriptome profiles of the biofilm-related genes of *V. parahaemolyticus* organisms under different sub-inhibitory stresses. The results proved that *V. parahaemolyticus* had a large growth difference under the two stresses, copper and CARB at 1/2 minimal inhibitory concentration (MIC), and it could form a stable biofilm under both stress conditions. The amount of biofilm formed under CARB stress was significantly higher than that of copper stress (*p* < 0.05). Based on the analysis of transcriptome sequencing results 323, 1,550, and 1,296 significantly differential expressed genes were identified in the three treatment groups namely 1/2 MIC CARB, Cu^2+^, and Cu^2+^+CARB. Through COG annotation, KEGG metabolic pathway analysis and gene expression analysis related to biofilm formation, the functional pathways of transcriptome changes affecting *V. parahaemolyticus* were different in the three treatment groups, and the CARB treatment group was significantly different from the other two groups. These differences indicated that the ABC transport system, two-component system and quorum sensing were all involved in the biofilm formation of the *V. parahaemolytic* by regulating flagellar motility, extracellular polysaccharides and extracellular polymer synthesis. Exploring the effects of different stress conditions on the transcriptome of *V. parahaemolyticus* could provide a basis for future research on the complex network system that regulates the formation of bacterial biofilms.

## 1. Introduction

*Vibrio parahaemolyticus*, a Gram-negative and halophilic human pathogen is widely distributed in the marine environments and frequently isolated from a variety of raw seafoods (Su and Liu, 2007). The consumption of raw or undercooked seafood contaminated with *V. parahaemolyticus* may lead to the development of acute gastroenteritis, accompanied by diarrhea, vomiting, nausea and abdominal cramps (Jo et al., 2020). With the steady development of the aquaculture industry, aquaculture farmers use different antibiotics to prevent and treat pathogenic bacterial infections. However, due to the excessive and irrational use of antibiotics, bacteria develop drug resistance and induce antibiotic resistance genes (Acharya and Wilson, 2019). Therefore, the problem of bacterial drug resistance in aquatic products should be paid more attention. As a nutrient, copper (Cu^2+^) is an essential trace element for microbial growth, but it adversely affects cell metabolism at high concentration (Zhou et al., 2018; Cai et al., 2019). Cu^2+^, a commonly used additive in aquaculture systems is often detected in environments such as rivers.

A biofilm permanently adheres to biotic and abiotic surfaces and comprises a complex aggregation of microorganisms that are surrounded by a matrix of extracellular polymeric substance (EPS) (Lin et al., 2020), a mode of life helps bacteria resist antibiotics and survive, and are ubiquitous in the natural environment (Kim et al., 2022). Food spoilage and foodborne infections are often caused by biofilm formation on food and food contact surfaces. Compared with free-living planktonic counterparts, bacterial biofilms could provide numerous benefits, including protection of cells from adverse environmental stresses, such as ultraviolet, antibiotics and other external pressures (Haque et al., 2017, 2021). Therefore, interfering with biofilm formation or stimulating its dissociation is an attractive strategy to combat bacterial infections and prevent their chronic development. Among them, the use of sub-concentration (below MICs, also referred to as sub-lethal levels) antibiotics is a critical reason for promoting the formation of bacterial biofilms (Zhang et al., 2018; Zuo et al., 2022). A study proved that exposure to sub-inhibitory concentrations of amoxicillin and tylosin increased the biofilm formation of *S. suis* (Zuo et al., 2022). *Lactobacillus plantarum* also showed similar results after the use of sub-inhibitory concentrations of gentamicin, kanamycin and streptomycin (George and Halami, 2017). Sub-inhibitory concentration antibiotics and heavy metals affect the formation of biofilm, and the same or different kinds of antibiotics or heavy metals on the formation of the same bacteria or different bacterial biofilm will produce different results. The current sub-inhibitory concentration of antibiotics on bacterial biofilm effect mechanism is not fully understood and need further research.

*Vibrio parahaemolyticus* senses the environment and expresses numerous genes, the products of which synergize to synthesize and secrete toxins that can cause acute disease on human. To understand the regulation of such adaptive response, mRNA transcripts must be mapped accurately (Al et al., 2021). The formation of bacterial biofilm is co-regulated by multiple genes and pathways, RNA-seq technology is a powerful tool for transcriptome analysis (Kong et al., 2022).

In our previous study, the presence of the antibiotic resistance gene carbenicillin (*CARB*) and the heavy metal tolerance gene (*CopA*) that can express copper transport ATPase were detected in VP35. Meanwhile, minimum inhibitory concentration (MIC) of Cu^2+^ and CARB were also detected. Therefore, in this study, Cu^2+^ and CARB were taken as the main research objects. Based on the sub-inhibitory concentration, the growth characteristics, biofilm formation and transcriptome expression of *V. parahaemolyticus* under different Cu^2+^ and CARB stress were studied to understand the effects of heavy metals and antibiotics on the formation of bacterial biofilm. In this present study, new strategies for removing biofilm will explore.

## 2. Materials and methods

### 2.1. Bacterial strain and culture conditions

VP35 was isolated from aquatic products in our laboratory. All strains stored at-80°C were recovered at TCBS agar plates. Then, one typical single colony was taken from the plate into 5 mL tryptic soy broth (TSB, Beijing Land Bridge Technology Co., Ltd., Beijing, China) supplemented with 2.5% NaCl (w/v) and cultured at 37°C and 200 rpm in a shaking incubator. After 6–8 h, the culture medium was centrifuged at 4,000× *g* and 4°C for 10 min. Finally, the bacterial solution was diluted to ~6 Log CFU/mL with sterilized saline (0.85% NaCl).

### 2.2. Antibiotic and heavy metal MIC determination

The minimal inhibitory concentration (MIC) was determined by broth dilution testing (microdilution) method using various concentrations of the antibiotic that was diluted serially in a microtiter plate (Wiegand et al., 2008). Briefly, 100 μL of CARB (Beijing Solarbio Science & Technology Co., Ltd., Beijing, China) that was serially diluted with TSB was mixed with 100 μL of bacterial solution (~6 Log CFU/mL) in 96-well plates, to give a total volume of 200 μL/well. After incubation at 37°C for 16–20 h, absorbance was checked using a microplate reader. To date, no standard method is available to measure bacterial susceptibility to heavy metals. According to the method described previously, the tolerance of the isolates to heavy metal was determined (He et al., 2016; Jiang et al., 2020). In a 96-well microtiter plate, *V. parahaemolyticus* was subjected to an array of heavy metal concentrations in TSB. The wells contained 100 μL of TSB plus heavy metals at concentrations ranging from 6.25 to 6,400 μg/mL for Cu^2+^ (Sangon Biotech Co., Ltd., Shanghai, China). The MIC was determined after 16–20 h of growth at 37°C. TSB without the culture inoculums was kept as negative control and medium that was inoculated with culture without antibiotic and heavy metal supplementation was used as the positive control. Each experiment was performed in triplicate.

### 2.3. Scanning electron microscopy

To evaluate the effects of CARB and Cu^2+^ on bacterial morphology, scanning electron microscopy (SEM) was performed. In this study, the MIC was 2 μg/mL of CARB and 1,600 μg/mL of Cu^2+^, and the inhibitory effect of biofilm was observed at sub-MIC that could not kill the bacteria. Thus, the concentrations used in this study were 1/4 MIC and 1/2 MIC. The prepared 2.5 mL samples (1/4, 1/2 MIC CARB, and Cu^2+^) were placed in 15 mL conical tubes containing 2.5 mL TSB of bacterial solution (~6 Log CFU/mL). Finally, they were mixed well using a vortex mixer and incubated at 37°C for 16 h. Afterwards bacterial suspension was centrifuged for 10 min at 3000 g. The resultant pellets were mixed with 1 mL glutaraldehyde (2.5%) for overnight at 4°C, after which the bacterial were washed several times with PBS, sequentially dehydrated with 30, 50, 70, 90, and 100% (v/v) ethanol, and 100% (v/v) ethanol was used twice for 10 min. The specimens were coated with gold for observation by SEM (SU5000, Hitachi High-Tech, Japan).

### 2.4. Growth experiment of *Vibrio parahaemolyticus*

A totol of 200 μL samples (1/2 MIC CARB, Cu^2+^ and Cu^2+^+CARB) were added to the plate of 100 microwells with *V. parahaemolyticus* (~6 Log CFU/mL) that was equipped with the Bioscreen C. OD_600nm_ values were read every 30 min at 37°C and the total operating time was 24 h. The data of *V. parahaemolyticus* was modeled using modified Gompertz model (Lianou and Koutsoumanis, 2011; Liu et al., 2016; Han et al., 2017; Yu et al., 2020).

The modified Gompertz model is as follows:


(1) $$y=Α+C\exp\left\{-\exp\left[\frac{\mu_{\max}}{Α}\left(\lambda -t\right)+1\right]\right\}$$

Where A is the initial inoculum of the strain (OD_600nm_); C represents the difference between the initial inoculum and the maximum number of strains (OD_600nm_); *μ*_max_ refers to the maximum specific growth rate (OD_600nm_/h); and *λ* marks the lag time duration (h).

### 2.5. Biofilm formation

This method was performed as previously described with some Modifications (Krom et al., 2007). A total of 500 μL of TSB medium with *V. parahaemolyticus* (~6 Log CFU/mL) and 500 μL CARB or Cu^2+^ were transferred into 24-well plates. Subsequently, the 24-well plates were incubated at 25°C statically to develop biofilm (Zhao et al., 2018). The biofilm was gently washed three times with 1 × PBS (Sangon Biotech Co., Ltd., Shanghai, China) and stained with 1 mL of 0.1% crystal violet (Sangon Biotech, Co., Ltd., Shanghai, China) for 30 min at 25°C, and then solubilized in 1 mL 95% ethanol for 30 min. The optical density of each well was measured at 600 nm.

### 2.6. Confocal laser scanning microscopy

To prove that CARB and Cu^2+^ influences biofilm formation of *V. parahaemolyticus*, the morphological identification of *V. parahaemolyticus* cells was performed by CLSM. First, biofilms were formed on each sample using the method that was already described in this study. Then, the samples were washed three times with 1 × PBS and fixed with 2.5% glutaraldehyde for 30 min at 4°C. All microscopy images were captured and acquired using the CLMS machine (LSM710, Carl Zeiss AG, Germany). The 40 × objective was used to monitor SYBR Green I (Sangon Biotech, Co., Ltd., Shanghai, China) fluorescence that was excited at 488 nm and emitted at 500-550 nm (Chen et al., 2020).

### 2.7. Whole genome transcriptome analysis

To prepare transcriptome samples, the overnight cultured *V. parahaemolyticus* was transferred into 15 ml conical tubes containing 1/2 MIC CARB, Cu^2+^ and Cu^2+^+CARB, and the cultures were continued to grow for 16–18 h at 37°C. The culture without CARB and Cu^2+^ addition was used as the control. Cells were collected and placed in liquid nitrogen for rapid cooling for 1 min for transcriptomic analysis. The data were analyzed on the online platform of Majorbio Cloud Platform.^1^

Gene Ontology (GO) functional annotation was performed using GO seq to describe molecular functions, cellular components, and biological processes that were associated with biofilm expression. Kyoto Encyclopedia of Genes and Genomes (KEGG) was used to identify the most important biochemical metabolic pathways and signal transduction pathways involved in differently expressed genes through major public databases. The function of the protein was predicted by the protein database Cluster of Orthologous Groups (COG) of proteins. *p* value and the false discovery rate (FDR ≤0.005) were used to correct the significance of gene differences.

Three genes were selected from transcriptome differential genes and validated by qRT-PCR (Supplementary Table S1). qRT-PCR was performed on a Real-Time PCR system (Roche LightCycler® 480). All qPCR reactions were performed in a total volume of 20 μL. Cycling parameters included an initial denaturation at 95°C for 15 min, followed by 40 cycles of 95°C for 10 s, 60°C for 20 s and primer extension at 72°C for 20 s. The changes in relative gene expression were calculated with the ^2–ΔΔCT^ method.

### 2.8. Statistical analysis

The experimental data were expressed as the mean ± standard deviation. In all tests, significant differences were set at *p* < 0.05, and all analyzes were performed using origin version 2022.

## 3. Results and discussion

### 3.1. Growth characteristics of biofilm

The analysis of growth curve usually uses mathematical model to fit bacterial growth, estimate various growth parameters of bacteria, and then to study the growth law of microorganisms. The Gompertz model is well known and widely used in many aspects of biology. It has been frequently used to describe the growth of animals and plants, as well as the number or volume of bacteria and cancer cells (Tjørve and Tjørve, 2017).

**Figure 1:**
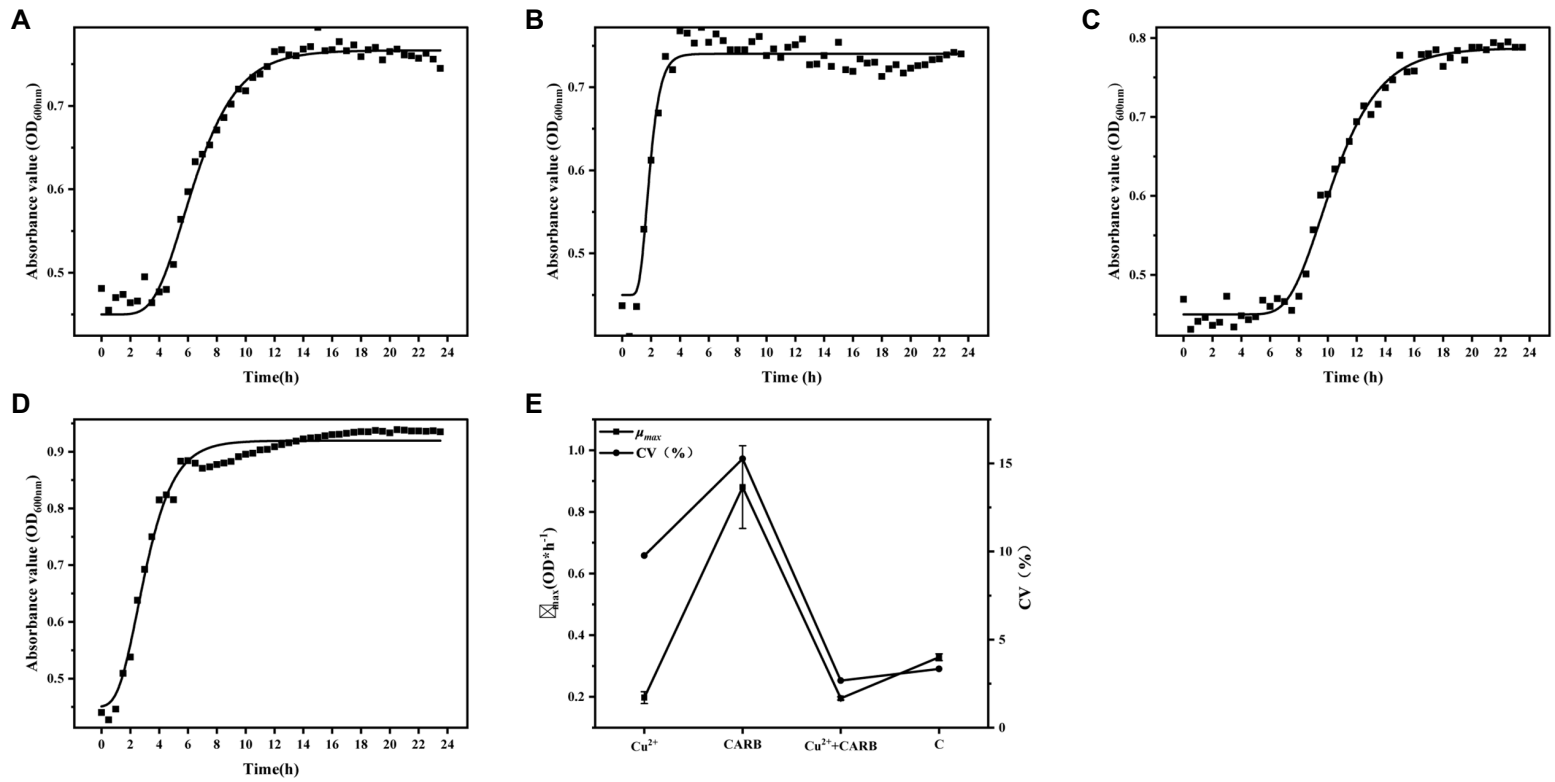
Determination of growth curve of *Vibrio parahaemolyticus* under different treatment conditions. **(A)** 1/2 MIC Cu^2+^ treatment, **(B)** 1/2 MIC CARB treatment, **(C)** 1/2 MIC Cu^2+^+CARB treatment, **(D)** control, **(E)** mean value curve and coefficient of variation curve (CV) of *μ_max_*.

The lag phase of 1/2 MIC Cu^2+^ treatment was longer than that of the control group, and 1/2 MIC CARB treatment was not significantly extended. The lag phase was the longest under 1/2 MIC Cu^2+^+CARB treatment, which showed that Cu^2+^ inhibit bacterial growth to some extent (Figures 1A-D). The exponential growth rate is one of the most important parameters, which represents the influence of gene and environment on bacterial growth dynamics and has great biological significance (Hall et al., 2014). The *μ_max_* was 0.20, 0.88, 0.19, and 0.33 under 1/2 MIC Cu^2+^, 1/2 MIC CARB, 1/2 MIC Cu^2+^+CARB and control, respectively. The results proved that CARB treatment *μ_max_* was maximized. It also proved the role of antibiotic in promoting the proliferation of pathogenic bacteria. The CV value of mean *μ_max_* was used to express the growth variability of *V. parahaemolyticus*. The CV values were 9.77, 15.27, 2.67, and 3.35%, under 1/2 MIC Cu^2+^, 1/2 MIC CARB, 1/2 MIC Cu^2+^+CARB and control, respectively (Figure 1E). Compared with Cu^2+^ treatment, there was greater growth variability under CARB treatment alone.

Our studies have revealed that both heavy metals and antibiotics can alter the physiological and biochemical responses of microorganisms, and adversely affected the number and growth of microbial populations. At the same time, microorganisms also adapted to the adverse environment through this change and rapidly accumulated mutant bacteria. In summary, it was found that under the common stress of Cu^2+^ and CARB, the growth amount, *μ_max_*, and CV value of *V. parahaemolyticus* greatly changed. Therefore, while monitoring antibiotic contamination in the environment, heavy metals also need to be paid more attention.

### 3.2. Morphology alteration

**Figure 2:**
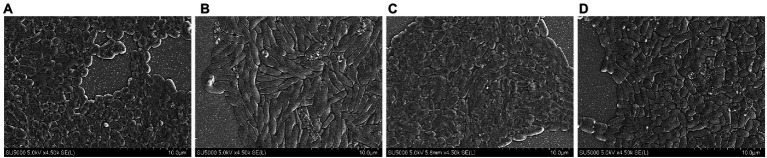
SEM of *Vibrio parahaemolyticus* under different treatment conditions. **(A)** 1/2 MIC Cu^2+^ treatment, **(B)** 1/2 MIC CARB treatment, **(C)** 1/2 MIC Cu^2+^+CARB treatment, **(D)** control.

The results of SEM can directly reflect the morphological changes of bacteria. According to the ultrastructural analysis of the surface of *V. parahaemolyticus* by SEM. The physiological and biochemical reactions of bacteria were different under heavy metal and antibiotic treatment. In negative control (without Cu^2+^ and CARB), the cells were plump and rod-shaped with a smooth surface characterized by SEM (Figure 2D). Most *V. parahaemolytic* cells have flat and rough edges, possibly because their cell walls were broken under Cu^2+^ stimulation (Figure 2A). Under CARB stimulation (Figure 2B), most *V. parahaemolyticus* cells were swelled and slender, and it was assumed that many biochemical reactions occurred *in vivo*, but they did not react violently under Cu^2+^ stimulation. Moreover, when the media was supplemented with Cu^2+^ and CARB, all these strains had both characteristics (Figure 2C). Therefore, the cell wall and cellular proteins in the *V. parahaemolyticus* cells were more vulnerable to be attacked by Cu^2+^ and CARB.

### 3.3. Biofilm formation

To visualize the growth differences of *V. parahaemolyticus* under different treatment conditions, the dynamic formation process of biofilm under different concentrations of antibiotic and heavy metal was monitored by crystal violet staining. With the extension of the incubation time, the overall value of OD_600nm_ showed a trend of first rising and then decreasing, which may be related to the biofilm formation law. The development of microbial biofilms involves five consecutive steps: (1) reversible attachment, (2) irreversible attachment, (3) microcolony formation, (4) biofilm maturation, and (5) dispersion (Ma et al., 2017; Hemati et al., 2020). At the initial stage, the adhesion ability of the bacterial membrane and the solid medium was weak, and a stable biological envelope structure was formed as the bacterial membrane became mature, and then the secretion of extracellular polysaccharides decreased, thus resulting in the decay, and shedding of the bacterial membrane.

**Figure 3:**
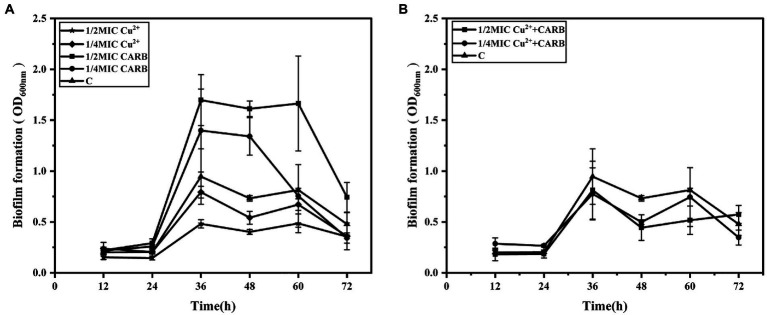
The dynamic process of biofilms formation by *V. parahaemolyticus* with time under different treatment conditions. **(A)** 1/2 and 1/4 MIC Cu^2+^ treatment, 1/2 and 1/4 MIC CARB treatment, **(B)** 1/2 and 1/4 MIC Cu^2+^+CARB treatment.

The crystal violet staining assay determined that 36 h was the optimal incubation time for the biofilm formation (OD_600nm_ = 0.95) and was selected in this study. Cu^2+^ or CARB treatment caused significant (*p* < 0.05) changes in the biofilm compared with the negative control (C). The 1/2 MIC Cu^2+^ treatment was obviously inhibited than the 1/4 MIC Cu^2+^ treatment, and the value of OD_600nm_ gradually decreased with the increase of Cu^2+^ concentration (Figure 3A). The results showed that Cu^2+^ could inhibit the biofilm formation of *V. parahaemolyticus*. However, CARB treatment alone promoted the biofilm formation of *V. parahaemolyticus*, and 1/2 MIC treatment had a stronger promotion effect than 1/4 MIC CARB treatment (Figure 3A), which also suggested that antibiotics with sub-inhibitory concentrations posed a risk of disease. When the two acted together, the dominant role was the inhibition of heavy metals (Figure 3B). The inhibitory effect of heavy metals can be used as a research focus in the future to prevent health risks caused by various pathogenic bacteria. To eradicate the biofilm, it is necessary to combine the addition of a variety of heavy metals to remove the result of removing the biofilm in future studies.

**Figure 4:**
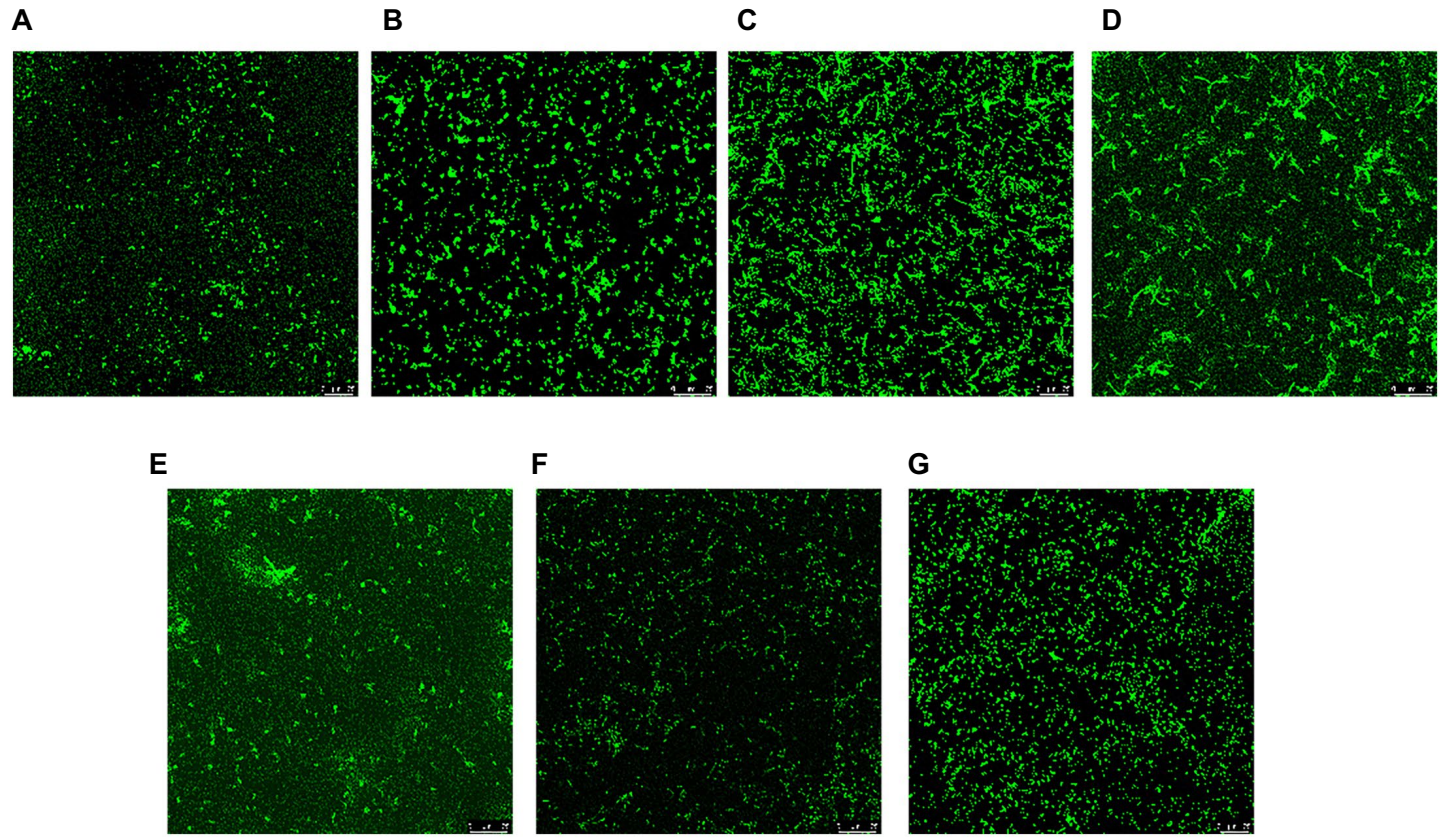
CLSM of *Vibrio parahaemolyticus* biofilm under different treatment conditions. **(A)** 1/2 MIC Cu^2+^ treatment, **(B)** 1/4 MIC Cu^2+^ treatment, **(C)** 1/2 MIC CARB treatment, **(D)** 1/4 MIC CARB treatment, **(E)** 1/2 MIC Cu^2+^+CARB treatment, **(F)** 1/4 MIC Cu^2+^+CARB treatment, **(G)** control.

CLSM was used to qualitatively visualize the three-dimensional structure of the biofilm at Cu^2+^ and/or CARB treatment. The negative control (C) of *V. parahaemolyticus* presented an intensively distributed biological architecture (Figure 4G). At Cu^2+^ treatment, the biofilm was obviously dispersed, and only a few single clusters of cells remained (Figures 4A,B). At CARB treatment, the fluorescence intensity of cells became stronger and aggregated into clusters, and it showed a slightly loose biofilm structure (Figures 4C,D). At Cu^2+^+CARB treatment, the fluorescence intensity of biofilm decreased significantly compared with control group (Figures 4E,F). Like the result of crystal violet staining, *V. parahaemolyticus* formed the biofilm at Cu^2+^ and/or CARB treatment.

### 3.4. Differential expressed gene analysis

**Figure 5:**
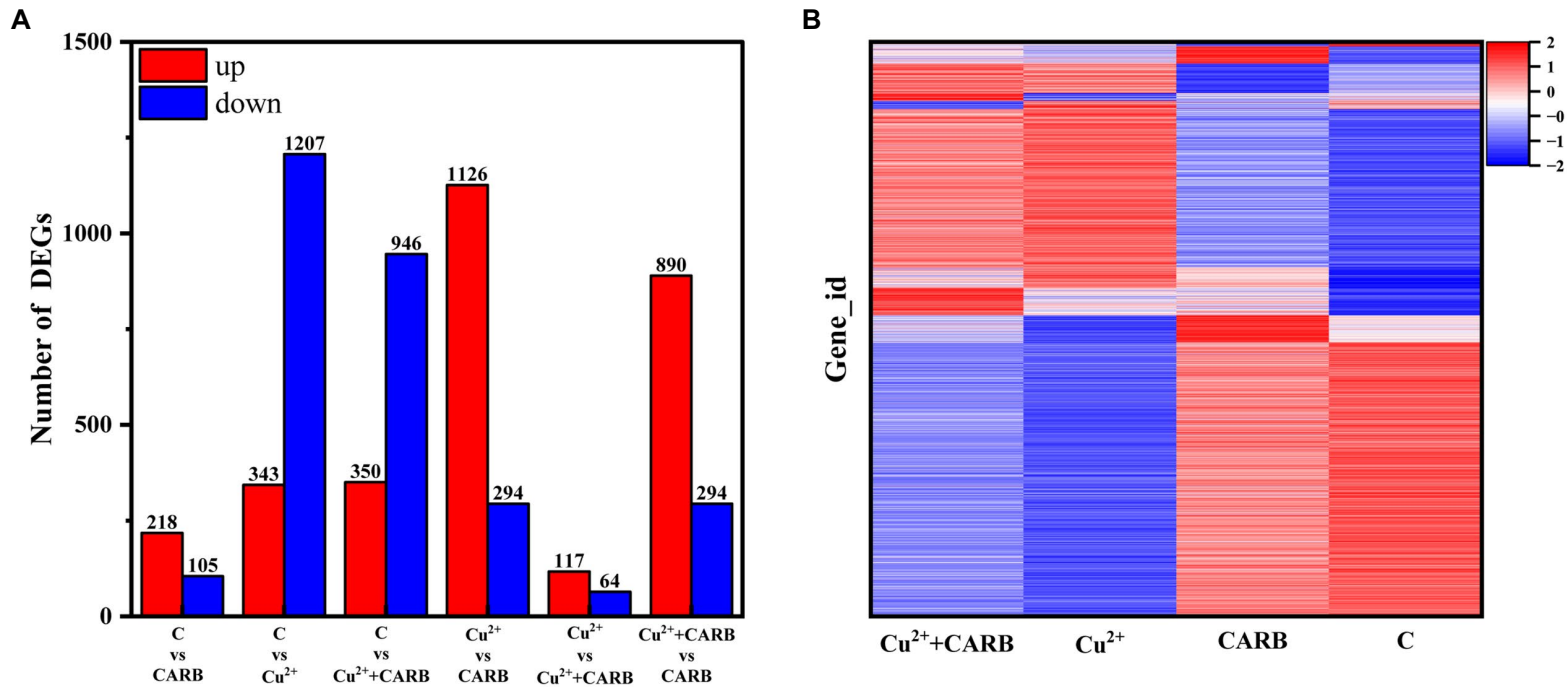
Differential expression analysis of experimental and control group. **(A)** Overall situation of differential genes in the three comparison groups. **(B)** Cluster diagrams. Different groups are represented horizontally. and different genes are represented vertically.

To explore the feedback regulation mechanism of *V. parahaemolyticus* resistance to Cu^2+^ and CARB stimulation, the transcriptional regulation of *V. parahaemolyticus* under Cu^2+^ or CARB stimulation was analyzed, and the *V. parahaemolyticus* without Cu^2+^ and/or CARB stimulation was used as a control. The statistics were calculated as *p*-adjust ≤0.05, FC = 2. The data showed that only the C vs. CARB treatment group up-regulated gene numbers (218) compared with Cu^2+^ or CARB treatment groups and was greater than the down-regulated group (105). In addition, there were 1,207 down-regulated genes in C vs. Cu^2+^ treatment, while only 1,126 genes were down-regulated in C vs. Cu^2+^+CARB treatment (Figure 5A). Moreover, the Cu^2+^ vs. CARB and Cu^2+^+CARB vs. CARB treatment group had a big difference in up and down-regulation genes, which also indicated that the effects of different treatment conditions on transcriptome expression (Figure 5B). To investigate the function of these DEGs under Cu^2+^ or CARB treatment during biofilm formation, a functional analysis was performed using GO and KEGG pathway terms.

**Figure 6:**
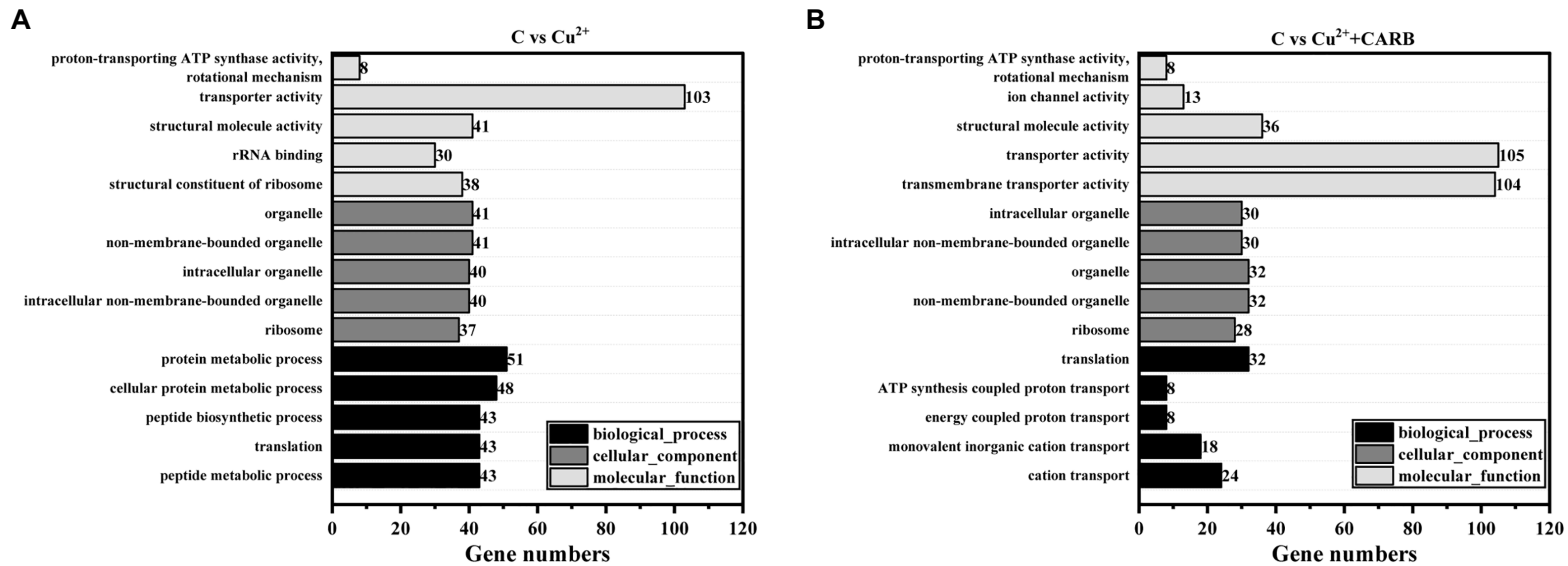
GO annotation information. **(A)** C vs. Cu^2+^, **(B)** C vs. Cu^2+^+CARB.

The differential genes of different treatment groups were annotated into three broad categories: biological process (BP), cellular component (CC), and molecular function (MF; Figure 6). The top five groups with significant differences were selected for each group. C vs. Cu^2+^ processing group comment entries include peptide metabolic process, translation, peptide biosynthetic process, cellular protein metabolic process, and protein metabolic process in BP, and ribosome, non-membrane-bounded organelle, organelle, intracellular non-membrane-bounded organelle, and intracellular organelle in CC, and structural constituent of ribosome, rRNA binding, structural molecule activity, transporter activity, proton-transporting ATP synthase activity, rotational mechanism in MF. Furthermore, C vs. Cu^2+^+CARB processing group comment entries are as follows: cation transport, monovalent inorganic cation transport, energy coupled proton transport, ATP synthesis coupled proton transport, and translation in BP; the CC category is the same as the first five of the C vs. Cu^2+^ treatment group; transmembrane transporter activity, transporter activity, structural molecule activity, ion channel activity, proton-transporting ATP synthase activity, and rotational mechanism in MF.

**Figure 7:**
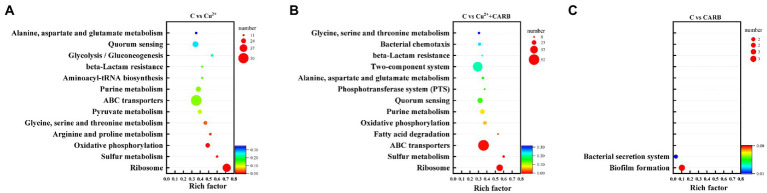
KEGG annotation information. **(A)** C vs. Cu^2+^, **(B)** C vs. Cu^2+^+CARB, **(C)** C vs. CARB.

Combined with the KEGG database, the ratio of pathway genes, the number of enriched genes, and the value of *p* were taken as indicators, the different metabolic pathways involving different genes were analyzed. In the comparison between the control and Cu^2+^ treatment group, the different genes were mainly involved in ribosome, sulfur metabolism, oxidative phosphorylation, arginine and proline metabolism, glycine, serine and threonine metabolism, pyruvate metabolism and ABC transport system. In the comparison between the control and Cu^2+^+CARB treatment group, the different genes were mainly involved in ribosome, sulfur metabolism, ABC transport system, fatty acid metabolism, oxidative phosphorylation and quorum sensing. However, in the comparison between the control and CARB treatment group, the differential genes were mainly enriched in the biofilm formation and the bacterial secretion system (Figure 7).

**Figure 8:**
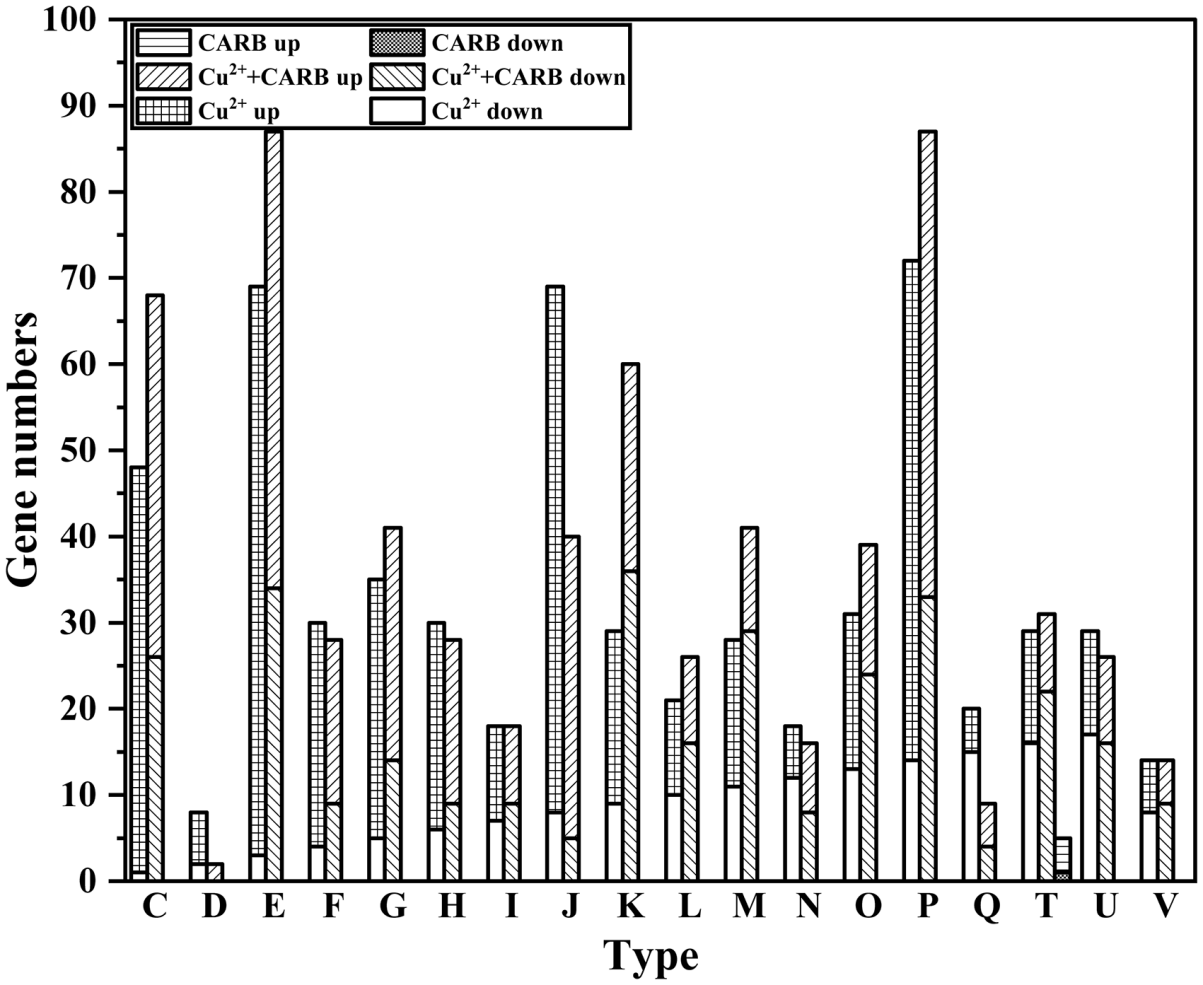
COG annotation information. C: Energy production and conversion. D: Cell cycle control, cell division, chromosome partitioning. E: Amino acid transport and metabolism. F: Nucleotide transport and metabolism. G: Carbohydrate transport and metabolism. H: Coenzyme transport and metabolism. I: Lipid transport and metabolism. J: Translation, ribosomal structure and biogenesis. K: Transcription. L: Replication, recombination and repair. M: Cell wall/membrane/envelope biogenesis. N: Cell motility. O: Posttranslational modification, protein turnover, chaperones. P: Inorganic ion transport and metabolism. Q: Secondary metabolites biosynthesis, transport and catabolism. T: Signal transduction mechanisms. U: Intracellular trafficking, secretion, and vesicular transport. V: Defense mechanisms.

Through the analysis of COG annotations, it was discovered that these significantly changed genes were mainly concentrated in the following categories excluding unknown function: amino acid transport and metabolism(E) (105, 87); Inorganic ion transport and metabolism(P) (90, 87); Energy production and conversion(C) (77, 68) under 1/2 MIC Cu^2+^ and 1/2 MIC Cu^2+^+CARB treatment. However, only 3 genes in the CARB treatment group were annotated in the signal transduction mechanisms(T; Figure 8). These results suggested that *V. parahaemolyticus* is resistant to Cu^2+^ and CARB throughout life activities.

### 3.5. Cu^2+^ and/or CARB affect/affects the expression of biofilm-forming genes

**Figure 9:**
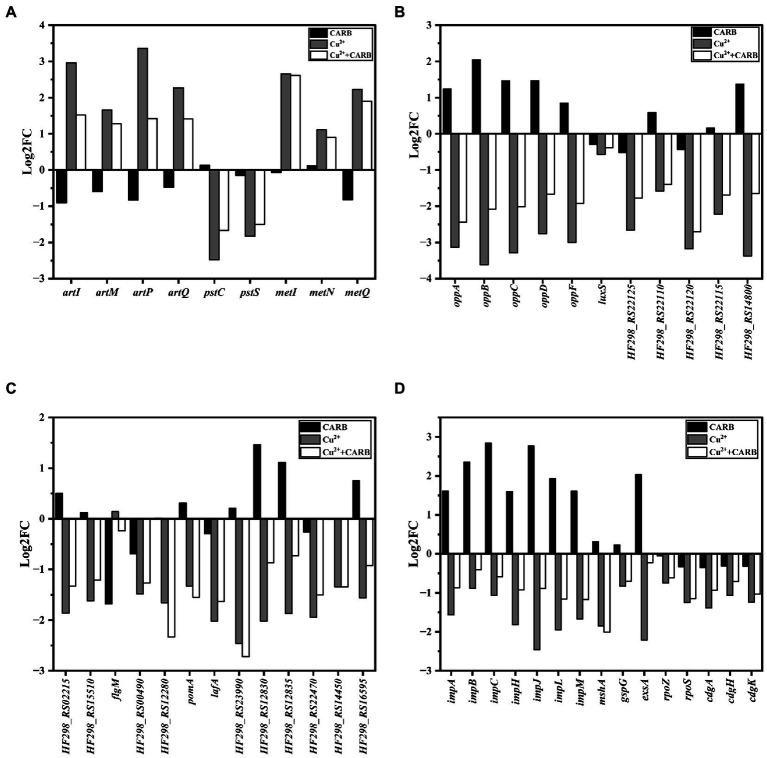
Differently expressed genes in biofilm related parts. **(A)** ABC transporters. **(B)** Quorum sensing systems. **(C)** Two-component systems. **(D)** others.

The genes *artI*, *artM*, *artP* and *artQ* were up-regulated by about 2.96, 1.66, 3.36 and 2.27 folds under 1/2 MIC Cu^2+^ treatment, and 1.52, 1.28, 1.42, and 1.41 folds under 1/2 MIC Cu^2+^+CARB treatment, which participated in arginine transport (Figure 9A). The expression of these genes could enhance their ability to transport amino acids, which indicated that the metabolic capacity of bacteria under the two treatment conditions of 1/2 MIC Cu^2+^ and 1/2 MIC Cu^2+^+CARB was stronger than that of 1/2 MIC CARB. Besides, a large amount of energy was required to provide energy supply with amino acid transport, which was not conducive to the formation of biofilm. Biofilm formation required cell adhesion, surface conditioning and EPS production, that were energetically expensive processes (Liu Z. et al., 2021). PstC and PstS proteins were involved in phosphate transport in bacteria, and the associated operons were *pstC* and *pstS*, respectively. The results of the previous studies have indicated that the active efflux system and biofilm formation are the main reasons for the development of multidrug resistance in bacteria. The transcriptome analysis results also confirmed that the genes *pstC* and *pstS* were down-regulated by about 2.48, 1.67 folds under 1/2 MIC Cu^2+^ treatment, and 1.83, and 1.50 folds under 1/2 MIC Cu^2+^+CARB treatment, respectively (Figure 9A), which mainly mediated bacterial resistance by influencing biofilm formation. At the same time, D-amino acids affect the biofilm formation by inhibiting the secretion of extracellular polysaccharides and extracellular proteins (Kolodkin-Gal et al., 2010; Aliashkevich et al., 2018). The D-methionine biosynthesis encoding genes *metI*, *metN* and *metQ* were up-regulated by about 2.66, 1.11, and 1.67 folds under 1/2 MIC Cu^2+^ treatment, and 2.91, 0.90, and 1.90 folds under 1/2 MIC Cu^2+^+CARB treatment, respectively (Figure 9A). It can be speculated that the large synthesis of D-amino acids led to a significant reduction in the amount of biofilm formation. In conclusion, it can be understood that the ABC transport system plays a role in the formation of biofilms and enhancement bacterial resistance.

The quorum sensing system (QS) consists of signals and receptors, quorum sensing regulators, and other controlled genes promotes the production of bacterial extracellular proteins and regulates the biofilm. The genes *oppA*, *oppB*, *oppC*, *oppD*, and *oppF* belong to the same operon opp regulation that can regulate the production of bacterial oligopeptide osmotase (Opp) (Li et al., 2020). They were up-regulated by about 1.24, 2.05, 1.46, 1.47, and 0.85 folds under 1/2 MIC CARB treatment, and down-regulated by about 1–4 folds under 1/2 MIC Cu^2+^ and 1/2 MIC Cu^2+^+CARB treatment (Figure 9B), which also corresponded to the amount of membrane production earlier. Opp is a family of ABC transporters that form transmembrane channels to participate in peptidoglycan metabolism, ingest oligopeptides into cells and protect the intracellular environment under stress. The *oppB* and *oppC* encoded proteins together formed a complete transmembrane protein. Most differential genes associated with QS were involved in encoding ABC transporters and ABC transporter osmotic enzymes, as well as various ATPases, which also indicated that QS can be involved in guiding a variety of ABC transporters to actively transport certain substrates into and out of cells to adapt to the biofilm formation. It has been pointed out that the deletion of luxS may directly or indirectly affect the receptors and downstream receptors that were regulated by AI-2 signaling molecules in *V. parahaemolyticus*, thereby changing the population behavior of bacteria and inhibiting the biofilm formation (Liu M. et al., 2021). The *luxS* was slightly down-regulated about 0–1 folds under all three stress conditions (Figure 9B). The gene *HF298_RS14800* was associated with extracellular proteins outside bacteria, and it was up-regulated about 1.37 folds, while down-regulated about 2.37 and 1.35 folds under 1/2 MIC Cu^2+^ and 1/2 MIC Cu^2+^+CARB treatment, respectively (Figure 9B). To form biofilm, cells aggregated and secreted many extracellular polysaccharides and extracellular proteins, that were firmly adhered to the surface of the object and play a decisive role in the strong adhesion of the biofilm, and also verified by different changes under different treatment conditions.

Two-component systems (TCSs) are a ubiquitous family of signal transduction pathways that enable bacteria to sense and respond to physical, chemical, and biological stimuli outside and inside the cell. Bacteria chemotaxis made bacteria tend to be more comfortable and environmental conditions and played an important role in many biological processes such as biofilm formation and host infection (Meng et al., 2020; Karmakar, 2021). In *V. parahaemolyticus* under 1/2 MIC Cu^2+^ and 1/2 MIC Cu^2+^+CARB treatment, *HF298_RS02215* and *HF298_RS15510* genes were down-regulated by about 1–2 folds (Figure 9C), and the encoded proteins were all methyl receptor chemotactic proteins. Methyl-accepting chemotaxis protein (MCP) can cross the cell membrane and sense chemical changes in the environment, and then induce bacterial adaptation to growth by signaling itself. The cytoplasmic proteins processed the sensory signals and then transmitted control signals to the flagellar motor to achieve the chemotactic movement (Zhang et al., 2019). The gene *flgM* is anti-σ factor, and negatively regulates the biosynthesis of flagellar filamentsplays. The gene was down-regulated by 1.68-folds under 1/2 MIC CARB treatment (Figure 9C). Conversely, 1/2 MIC Cu^2+^ and 1/2 MIC Cu^2+^+CARB treatment were slightly improved. The genes *HF298_RS00490*, *HF298_RS12280*, *pomA*, and *lafA* related to flagellin synthesis were down-regulated by about 1.48, 1.66, 1.33, and 2.02 folds under 1/2 MIC Cu^2+^ treatment, and 1.27, 2.33, 1.55, and 1.63 folds under 1/2 MIC Cu^2+^+CARB treatment, respectively (Figure 9C). It was speculated that MCP interacts with flagellin to affect bacterial attachment and biofilm formation.

*HF298_RS12830* and *HF298_RS12835* genes were up-regulated by about 1.46 and 1.11 folds under 1/2 MIC CARB treatment, and down-regulated by about 1–3 folds under 1/2 MIC Cu^2+^ and 1/2 MIC Cu^2+^+CARB treatment (Figure 9C). Their encoded proteins were TRAP transporter protein substrate binding protein and TRAP transporter protein small permease, and used ion electrochemical gradients to provide energy for solute uptake (Ng et al., 2019). From the transcriptome results, it can be guessed that the encoded proteins not only cooperate with TCS, but also positively regulate the formation of biofilm. *HF298_RS22470* and *HF298_RS14450* genes belong to the OmpR protein family of TCS and are responsible for the transport of phosphate groups. Furthermore, they were up-regulated by about 0–1 folds under 1/2 MIC CARB treatment, and down-regulated by about 1–2 folds under 1/2 MIC Cu^2+^ and 1/2 MIC Cu^2+^+CARB treatment (Figure 9C). As a regulatory protein in the EnvZ/OmpR-TCS, the outer membrane protein, OmpR can positively regulate V.parahaemolyticus biofilm formation (Zuberi et al., 2022). In response to external osmotic stress, EnvZ transmited a signal to OmpR with transcriptional activity by means of a phosphate group, thereby regulating the transcription of outer membrane porin and participating in the regulation of biofilm formation.

The genes *impA*, *impB*, *impC*, *impH*, *impJ* and *impM* belong to T6SS2 that mainly mediates its adhesion to host cells and is also related to the formation of biofilm formation. In *V. parahaemolyticus* stimulated by 1/2 MIC CARB, *impA*, *impB*, *impC*, *impH*, *impJ*, and *impM* were mostly up-regulated at a fold of 1–3 folds, while down-regulated under 1/2 MIC Cu^2+^ and 1/2 MIC Cu^2+^+CARB treatment (Figure 9D). Biofilm formation was caused by surface attachment that is controlled by MshA pilus (Jones et al., 2015). These fimbriae were assembled by polymerization of the MshA subunit. The gene mshA related to fimbriae formation was down-regulated 1.85 and 2.01 folds under 1/2 MIC Cu^2+^ and 1/2 MIC Cu^2+^+CARB treatment, respectively (Figure 9D). In Gram-negative bacteria, type II secretion systems (T2SS) assemble inner membrane proteins of the major pseudopilin GspG family into periplasmic filaments, which could drive protein secretion in a piston like manner (Cisneros et al., 2012; Michel-Souzy et al., 2018). The gene *gspG* encoding transmembrane proteins in T2SS (Lory, 1998) was down-regulated 0.5–1 folds under 1/2 MIC Cu^2+^ and 1/2 MIC Cu^2+^+CARB treatment (Figure 9D). However, when 1/2 MIC CARB treatment, the gene change was not obvious. It was speculated that it may be positively correlated with the biofilm formation, but the correlation was not clear.

*Vibrio parahaemolyticus* has two sets of T3SSs that are located on chromosome 1 (T3SS1) and chromosome 2 (T3SS2). The T3SS1 system mediates bacterial cytotoxicity by inducing programmed death or autophagy of host cells. Regulatory protein ExsA can directly binds to the promoter region of the T3SS1 gene cluster to induce T3SS1 expression (Li et al., 2019). Under 1/2 MIC CARB treatment, the gene *exsA* was up-regulated 2.04 folds, and it was down-regulated 2.22 folds under 1/2 MIC Cu^2+^ treatment, while the effects were not obvious when combined treatment (Figure 9D). Previous studies of *V. parahaemolyticus* reported that biofilm production and T3SS1 genes expression are inversely regulated or no correlation (Gode-Potratz and McCarter, 2011; Calder et al., 2014). Yet despite this connection, we found the opposite, but it is also possible that this correlation was weakened under different induction conditions, which can be further studied in the future. The gene *rpoS* is responsible for regulating the formation of RNA polymerase (Feng et al., 2021), and was down-regulated 1.25 and 1.15 folds under 1/2 MIC Cu^2+^ and 1/2 MIC Cu^2+^+CARB treatment (Figure 9D). It has been pointed out that the culture stress of the *rpoS* deletion and the *Shewanella baltica* could lead to a decrease in the secretion capacity of extracellular polysaccharides, thus resulting in a decrease in the formation of biofilm (Feng et al., 2021). A key regulator of the transition between a motile state and a biofilm state is the second messenger cyclic-dimeric guanosine monophosphate (c-di-GMP) (Wu et al., 2020). In addition, the genes *cdgA*, *cdgH*, and *cdgK* are responsible for encoding C-di-GMP that controls the formation of biofilm (Kearns, 2019), but all the three treatments down-regulated the *cdg* operon, of which the 1/2 MIC CARB treatment down-regulation factor was less than 0.5 folds that was significantly lower than the other two treatments (Figure 9D).

**Figure 10:**
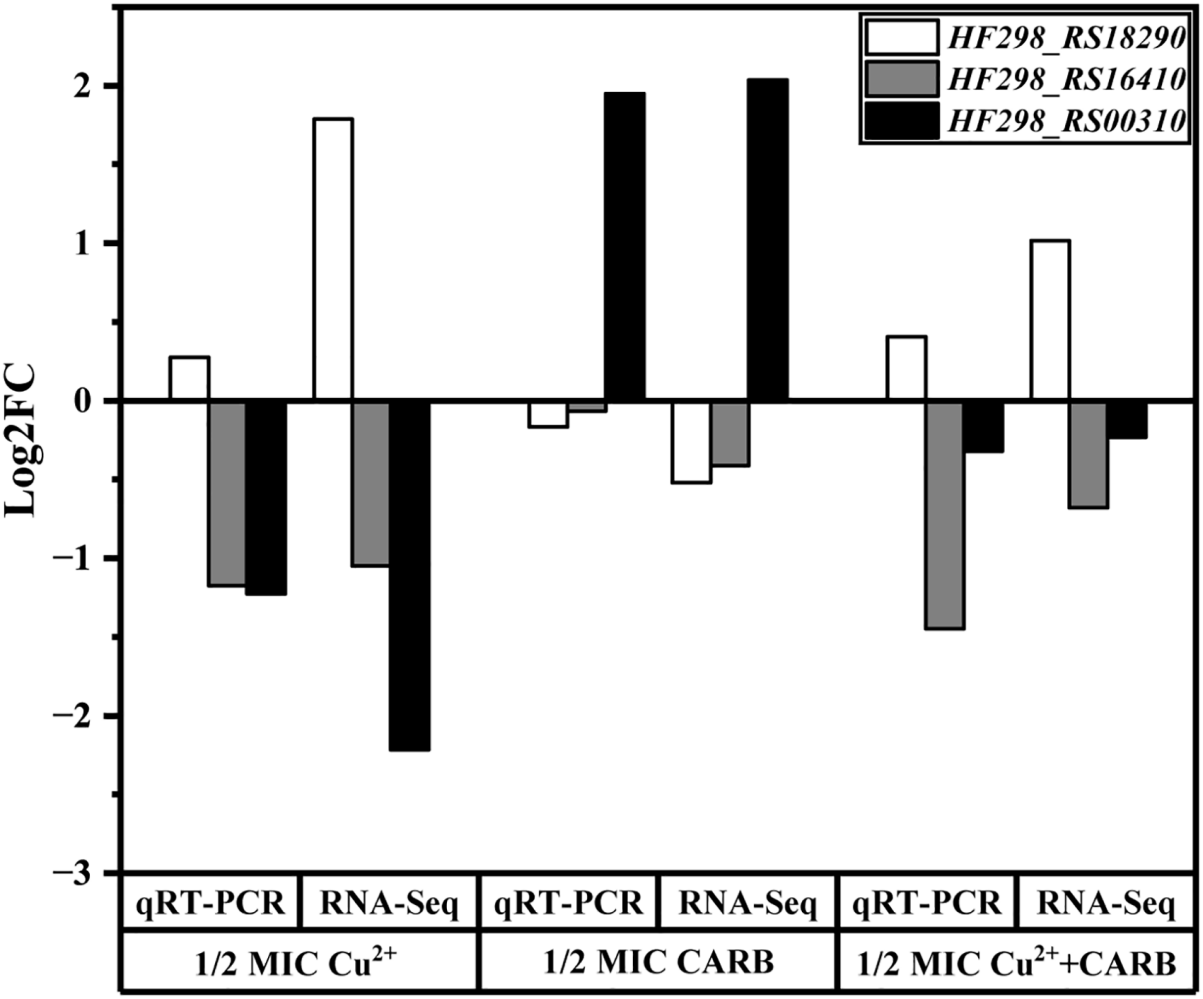
Analysis of differential genes expression levels between RNA-Seq and qRT-PCR.

Three genes were selected from transcriptome differential genes and validated by qRT-PCR, which was consistent with the trend of transcriptome results and demonstrated reliable transcriptome data. The result is shown in the following figure. In addition, we have also added supplements data to the article (Figure 10).

## 4. Conclusion

In conclusion, the results of the present study indicated that under different treatment conditions, *V. parahaemolyticus* showed different growth. Moreover, Cu^2+^ inhibited the formation of the biofilm, and conversely, CARB was significantly promoted, which was also demonstrated by the transcriptome analyzes. Through Transcriptome sequencing, it was found that Cu^2+^ inhibited biofilm formation mainly by controlling flagellar motility, extracellular polysaccharides and extracellular polymer synthesis. Under CARB treatment, just the opposite. Cu^2+^ and CARB treatments were posed on biofilm formation, of which the inhibition of Cu^2+^ took the dominate side. Exploring the effects of different stress conditions on the transcriptome of *V. parahaemolyticus* could provide a basis for future research on the complex network system that regulates the formation of bacterial biofilms. Our experiments have found that the effects of copper and carbenicillin have different effects on the alteration of biofilms, and have demonstrated this through transcriptome sequencing. Of course, there are some limitations to our experiment. We only selected one kind of metal and antibiotic to complete our experimental study, which may not be enough to represent all kinds of heavy metals and antibiotics. Most importantly, future studies should focus on the interaction mechanism between biofilms and heavy metals and the development of effective heavy metal-based biofilm removal technologies.

## Data availability statement

The original contributions presented in the study are included in the article/Supplementary material, further inquiries can be directed to the corresponding author.

## Author contributions

JX and HZ performed the lab experiments and wrote the main manuscript text. YL and HL analyzed the data and contributed to reagents, materials, and analysis tools. YP and YZ supervised the research. QX designed the experiments and revised the manuscript. All authors contributed to the article and approved the submitted version.

## Funding

This study was supported by the Shanghai Agriculture Applied Technology Development Program (Grant No. X20210302) and the National Natural Science Foundation of China (31972188).

## Conflict of interest

The authors declare that the research was conducted in the absence of any commercial or financial relationships that could be construed as a potential conflict of interest.

## Publisher’s note

All claims expressed in this article are solely those of the authors and do not necessarily represent those of their affiliated organizations, or those of the publisher, the editors and the reviewers. Any product that may be evaluated in this article, or claim that may be made by its manufacturer, is not guaranteed or endorsed by the publisher.

## Supplementary material

The Supplementary material for this article can be found online at: https://www.frontiersin.org/articles/10.3389/fmicb.2023.1128166/full#supplementary-material

## Footnotes

^1^ www.majorbio.com
