# Supplementary material for: Transcriptome analysis of the biofilm formation mechanism of Vibrio parahaemolyticus under the sub-inhibitory concentrations of copper and carbenicillin
Source: Front Microbiol. 2023 Mar 2;14:1128166. doi: 10.3389/fmicb.2023.1128166 (PMC10018186; doi:10.3389/fmicb.2023.1128166)
Supplement: Supplementary file 1 [file Table_1.DOCX]

Supplementary Material

Transcriptome analysis of the biofilm formation mechanism of *Vibrio parahaemolyticus* under the sub-inhibitory concentrations of copper and carbenicillin

Jiaying Xie^1†^, Hongmin Zhang^1†^, Yinhui Li^1^, Hao Li^1^, Yingjie Pan^1,2,3^, Yong Zhao^1,2,3†*^, Qingchao Xie^1,2†*^

^1^College of Food Science and Technology, Shanghai Ocean University, Shanghai, China

^2^Laboratory of Quality & Safety Risk Assessment for Aquatic Product on Storage and Preservation (Shanghai), Ministry of Agriculture and Rural Affairs, Shanghai, China

^3^Shanghai Engineering Research Center of Aquatic Product Processing & Preservation, Shanghai, China

*** Correspondence:**Yong Zhao
[yzhao@shou.edu.cn](mailto:yzhao@shou.edu.cn)

Qingchao Xie
[qcxie@shou.edu.cn](mailto:qcxie@shou.edu.cn)

# Supplementary Table

**Table 1.** Primers used in the study.

| Gene | Primer name | Sequence of primers (5′-3′) | Annealing temperature(°C) | Product size (bp) |
| --- | --- | --- | --- | --- |
| *HF298_RS18290* | FW | CGGTGAGTTGCTGTTGTTGG | 59.97 | 121 |
|  | RV | TCAACCGCTCATCGTCTGTC | 60.11 |  |
| *HF298_RS16410* | FW | CAGGACAAGCCGATACCCAA | 59.75 | 124 |
|  | RV | CCAAACCGTAAAAGCAGCGA | 59.41 |  |
| *HF298_RS00310* | FW | ATAGAGGACTTCGCCCCCTT | 60.03 | 105 |
|  | RV | ATGAAAACGCTGGAGGAGCA | 59.96 |  |
